# User Participation and Engagement With the See Me Smoke-Free mHealth App: Prospective Feasibility Trial

**DOI:** 10.2196/mhealth.7900

**Published:** 2017-10-09

**Authors:** Chris A Schmidt, James K Romine, Melanie L Bell, Julie Armin, Judith S Gordon

**Affiliations:** ^1^ College of Public Health University of Arizona Tucson, AZ United States; ^2^ Family and Community Medicine University of Arizona Tucson, AZ United States; ^3^ College of Nursing University of Arizona Tucson, AZ United States

**Keywords:** mHealth, mobile health, intervention, smoking, diet, physical activity

## Abstract

**Background:**

The See Me Smoke-Free (SMSF) mobile health (mHealth) app was developed to help women quit smoking by targeting concerns about body weight, body image, and self-efficacy through cognitive behavioral techniques and guided imagery audio files addressing smoking, diet, and physical activity. A feasibility trial found associations between SMSF usage and positive treatment outcomes. This paper reports a detailed exploration of program use among eligible individuals consenting to study participation and completing the baseline survey (*participants*) and ineligible or nonconsenting app installers (*nonparticipants*), as well as the relationship between program use and treatment outcomes.

**Objective:**

The aim of this study was to determine whether (1) participants were more likely to set quit dates, be current smokers, and report higher levels of smoking at baseline than nonparticipants; (2) participants opened the app and listened to audio files more frequently than nonparticipants; and (3) participants with more app usage had a higher likelihood of self-reported smoking abstinence at follow up.

**Methods:**

The SMSF feasibility trial was a single arm, within-subjects, prospective cohort study with assessments at baseline and 30 and 90 days post enrollment. The SMSF app was deployed on the Google Play Store for download, and basic profile characteristics were obtained for all app installers. Additional variables were assessed for study participants. Participants were prompted to use the app daily during study participation. Crude differences in baseline characteristics between trial participants and nonparticipants were evaluated using *t* tests (continuous variables) and Fisher exact tests (categorical variables). Exact Poisson tests were used to assess group-level differences in mean usage rates over the full study period using aggregate Google Analytics data on participation and usage. Negative binomial regression models were used to estimate associations of app usage with participant baseline characteristics after adjustment for putative confounders. Associations between app usage and self-reported smoking abstinence were assessed using separate logistic regression models for each outcome measure.

**Results:**

Participants (n=151) were more likely than nonparticipants (n=96) to report female gender (*P*<.02) and smoking in the 30 days before enrollment (*P*<.001). Participants and nonparticipants opened the app and updated quit dates at the same average rate (rate ratio [RR] 0.98; 95% CI 0.92-1.04; *P*=.43), but participants started audio files (RR 1.07; 95% CI 1.00-1.13; *P*<.04) and completed audio files (RR 1.11; 95% CI 1.03-1.18; *P*<.003) at significantly higher rates than nonparticipants. Higher app usage among participants was positively associated with some smoking cessation outcomes.

**Conclusions:**

This study suggests potential efficacy of the SMSF app, as increased usage was generally associated with higher self-reported smoking abstinence. A planned randomized controlled trial will assess the SMSF app’s efficacy as an intervention tool to help women quit smoking.

## Introduction

Mobile technology offers new tools for delivering cost-effective and scalable health interventions to diverse populations [1]. Smoking cessation is an important focus for mobile health (mHealth) initiatives, considering the heavy burden of tobacco-related morbidity and mortality [2] and the exponential increase in access to mobile phone devices [3]. A recent meta-analysis suggests that mHealth cessation programs result in increased quit rates among participants [4], although the degree of usage needed to attain desired health outcomes is often uncertain [5]. Furthermore, the degree of usage may be influenced by sociodemographic factors, age, psychological traits, and other user characteristics [6]. These factors should be considered when evaluating the effectiveness of mHealth interventions, such as the recently-developed See Me Smoke-Free (SMSF) multi-behavioral smoking cessation program.

The SMSF mHealth app was developed to help women quit smoking by targeting concerns about body weight, body image, and self-efficacy through the use of guided imagery audio files that address smoking, diet, and exercise [7]. The development of SMSF has been reported elsewhere [8]. Gordon et al [9] assessed and presented feasibility outcomes and exploratory analyses of program impact and identified possible associations between app usage and both smoking and dietary behavioral change. As program use was associated with positive treatment outcomes in the pilot study [9], further exploration of app use data was warranted. This study expands our understanding of feasibility outcomes of SMSF by exploring the representativeness of our sample versus all users of the app. As others have noted, analysis of utilization by user characteristics may have implications for program enhancements and dissemination of the app to appropriate populations [10]. Moreover, considering the broad array of smoking cessation programs on the market, feature-level analyses may point to elements that predict desired outcomes such as smoking abstinence [11].

SMSF was deployed to the Google Play Store and available for use by the public. All those who downloaded the app were invited to enroll in the study, and approximately half of all users enrolled as participants [9]. We collected profile data and Google Analytics usage data for all users and outcome data for study participants. *Participants* were defined as eligible individuals who consented to study participation and completed the baseline survey, whereas *nonparticipants* were ineligible or nonconsenting app installers. In this paper, we report on our analysis of these data to explore program use among all those who downloaded the app, as well as the relationship between program use and treatment outcomes. Our goals were to (1) compare baseline characteristics of SMSF trial participants with people who downloaded and used the app but did not participate in the study (nonparticipants), (2) estimate associations between participant baseline characteristics and app usage, (3) evaluate whether trial participation is associated with higher app usage, and (4) assess associations between app usage and smoking cessation among participants. We hypothesized that the participants are more likely to set quit dates, be current smokers, and report higher levels of smoking at baseline than nonparticipants; that participants open the app and listen to audio files more frequently than nonparticipants; and that participants with more app usage have higher likelihood of smoking cessation at follow-up.

We were interested in exploring potential differences between participants and nonparticipants for two main reasons, which are as follows: (1) to understand how app users who enroll in a research study compare with those who do not and (2) to determine whether those who did not meet our initial criteria would still use the app and find it potentially useful. Although SMSF was not marketed to nonsmokers, it is possible that individuals who had stopped smoking for 30 days or more might use the program to prevent relapse. It is also possible that nonsmoking individuals may have downloaded the app to consider whether it was something that they would want to recommend to a friend or family member who smoked.

Reporting follows the consolidated standards of reporting trials (CONSORT) guidelines for feasibility trials [12].

## Methods

### Study Design

The SMSF feasibility trial was a single arm, within-subjects, prospective cohort study (ClinicalTrials.gov NCT02972515). Details of this trial, including eligibility criteria, have been described elsewhere [7]. Briefly, the SMSF app delivered five imagery audio files, one of which was a general introduction to guided imagery [9]. Three files were designed to target specific behaviors—smoking cessation, physical activity, and fruit and vegetable consumption—and another addressed general wellness, positive body image, and self-efficacy [9]. Study participants were recruited via news stories and social media postings. Individuals who installed the app and completed app setup during the recruitment period (April 1, 2015 to July 31, 2015) were invited to participate in the feasibility study. Individuals were eligible for participation if they identified as female, were at least 18 years old, smoked in the last 30 days, lived in the United States, used an Android phone, agreed to use the app “most days for 30 days,” spoke English, and had a valid email address [7]. After eligibility screening, eligible individuals consenting to study participation and completing the baseline survey were enrolled (*participants*). Both study participants and ineligible or nonconsenting app installers (*nonparticipants*) could receive the study intervention, which was defined as the degree of app usage. Eligible nonparticipants were not asked to explain why they declined to participate in the study. Outcomes included app usage (as defined by number of times participants listened to guided imagery audio files, number of times participants answered the daily questions, and so on) for all users and smoking status of participants at follow-up. Incentives of US $25 were provided to participants upon completing each 30- and 90-day assessment. Users could receive app-based awards if they met their goals for a week, but they did not receive monetary compensation.

Basic profile characteristics (eg, demographics and tobacco use) were obtained for all app installers at registration, and additional baseline variables were assessed for participants. Participant outcomes were evaluated at 30 and 90 days post enrollment. A full description of the assessment questionnaire was provided in Gordon et al [9]. App usage was tracked continuously during the trial (April 1, 2015 to October 15, 2015) via app-based analytics (participants only) and Google Analytics (all installers). Participants were asked to set a quit date during app setup but were able to defer setting the date and return to the quit date tool to set a date. Quit dates were recorded in the tracking system, and awards were given based on the quit date. The system’s motivational push messages were tailored to a user's stage in the quitting process. The app delivered daily prompts via push notifications to participants to report their smoking status (“Overall, please rate your cravings today” and “Did you smoke today, even a puff?”) and reminders to listen to guided imagery audio files at user-specified times. Four 5-min guided imagery audio files focused on smoking cessation, eating well, increasing physical activity, and maintaining general well-being were delivered consecutively over 4 weeks. A complete description of program content, including prompt texts, is provided in a separate paper [9].

The app was identical for participants and nonparticipants, and both groups were prompted to access app features and complete questions daily; however, only participants were asked to complete the baseline and 30- and 90-day surveys. Participants were required to use the app for at least 30 days during study participation. Staff attempted to contact participants who did not use the app for 2 consecutive weeks, and participants who opted to remain and subsequently used the app were retained. If participants did not use the app for 2 consecutive weeks (14 days) during the first 30 days and the staff was not able to contact them, they were dropped for inactivity. Participants who completed the study were considered *full participants*, whereas those who dropped out or were withdrawn were considered *partial participants*. Data from partial participants were not retained or included in the analysis, though baseline variables were previously reported to not differ significantly between partial and full participants [9].

### Study Outcomes and Covariates

**Figure 1:**
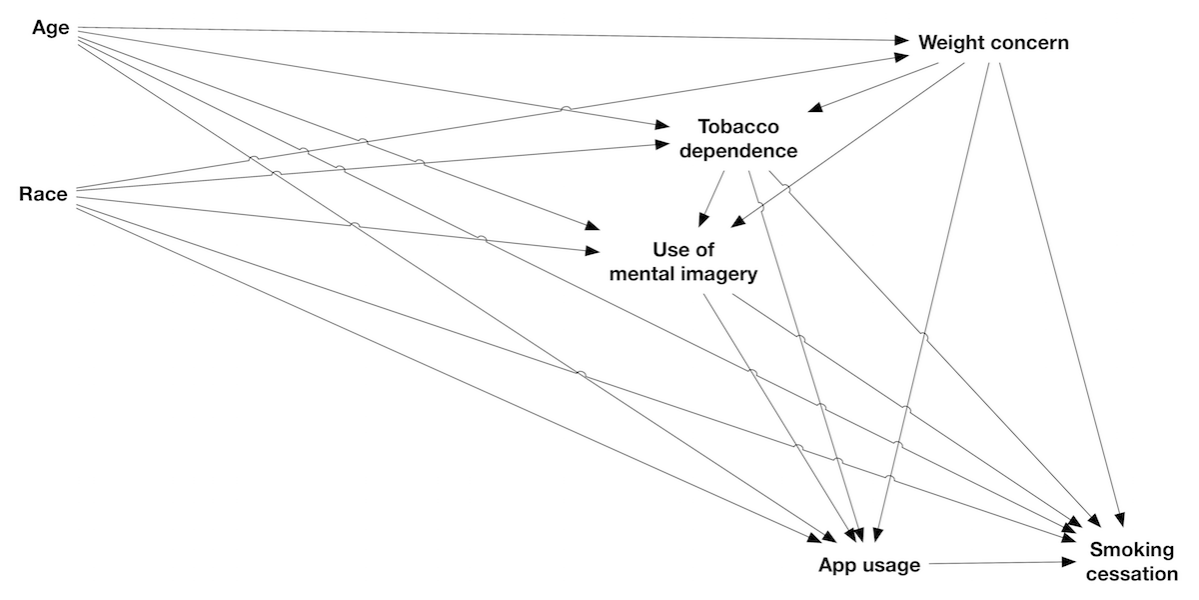
Directed acyclic graph showing hypothesized relationships among principal exposure, confounder and outcome variables (see text for details of which variables filled these roles in each analysis). App usage and smoking cessation each consist of several measures and are displayed here in a simplified form.

Outcomes assessed by this analysis consisted of app usage and smoking cessation indicators. We hypothesized an a priori conceptual relationship model for exposure, outcome, and putative confounder variables (Figure 1). Age and race are commonly considered likely confounders of associations in epidemiological studies. Weight concern was considered likely to influence tobacco dependence [7], whereas both weight concern and tobacco dependence were considered probable influences on motivation to use mental imagery or the app. Guided by this model, separate analyses were used to examine usage as an outcome of demographic and behavioral variables and as an exposure associated with smoking cessation outcomes.

Google Analytics data represent aggregate counts (for participants and nonparticipants separately) of times opening the app, updating quit dates, and starting or completing guided imagery audio files. Outcomes were measured as unique screen views per app session over the entire study period. Audio file data were combined to yield aggregate measures across all audio files. The app collected data in a separate database on audio files completed and days answering questions for each participant.

Completion rates for daily individual questions were recorded; however, no monetary reward or compensation was offered for completing the daily assessments. Smoking data were collected at 30 and 90 days and consisted of 7-, 30-, and 90-day self-reported smoking abstinence.

Characteristics collected during app registration for all installers included gender, smoking status in previous 30 days (yes or no), desire to set a quit date (yes or no), number of cigarettes smoked on a typical day, amount paid for a pack of cigarettes, and source of referral to SMSF (from a coded list). Variables with which to address associations between participant characteristics and app usage were sourced from the baseline questionnaire and included age (in years, as 2015 minus reported birth year), race (dichotomized to white or non-white because of small samples for non-white races), tobacco dependence (time to smoking after waking, four categories from <5 min to >60 min), concern about weight gain (low to high willingness to gain 1-5 lb after quitting smoking, on an integer scale of 1 to 5; modeled as a continuous variable given evident linearity with outcome measures), and use of mental imagery in the previous week (never to greater than 30 min, on an integer scale of 1 to 5).

### Statistical Methods

All statistical analyses were performed in R version 3.3.1 (R Core Group) [13], and a type I error rate of 0.05 was prespecified for all tests of significance, which were two-sided. Crude differences in baseline characteristics between participants and nonparticipants were evaluated using two-sample *t* tests for continuous variables and Fisher exact tests for categorical variables. Exact Poisson tests were used to assess group-level differences in mean usage rates over the full study period using aggregate Google Analytics data on participation and usage.

Usage variables from app-based analytics displayed right-skewed distributions, supporting the use of negative binomial or Poisson regression models to estimate associations of app usage (days answering questions and number of audio files completed) with participant baseline characteristics. Negative binomial models were compared with equivalent Poisson models using likelihood ratio tests (LRTs) to assess improvement in model fit with the former models. Individual models estimated associations of each outcome variable with age, race, tobacco dependence, weight concern, and use of mental imagery. Adjusted models were specified from putative confounding relationships (Figure 1). Models for tobacco dependence included weight concern as a covariate, and models for mental imagery included weight concern and tobacco dependence. All models included race and age as possible confounders. Age was treated as a continuous variable with either linear or nonlinear terms (three-knot restricted cubic splines) selected for each model via LRTs. Observations with Cook *d* above 4/n (where n is the sample size for the analysis) within a given adjusted model were removed and the model rerun to assess the sensitivity of results to these potentially influential observations.

Associations between app usage and smoking cessation among individual full participants were assessed using separate logistic regression models for each self-reported smoking abstinence outcome. Primary exposure variables were modeled separately and consisted of days answering questions and audio files completed. Usage data for the first 30 days of participation were summed for each individual. Age (linear), race, weight concern, mental imagery, and tobacco dependence were included as potential confounders. Assumptions of linearity in log-odds were assessed, and log transformation and nonlinear modeling of exposure variables were evaluated for improvement of linearity and model fit. Discriminatory abilities of final models were evaluated using calculated *C* indices. Identification of influential points and handling of missing data were as described previously for negative binomial models.

## Results

### Participant Recruitment and Characteristics

**Figure 2:**
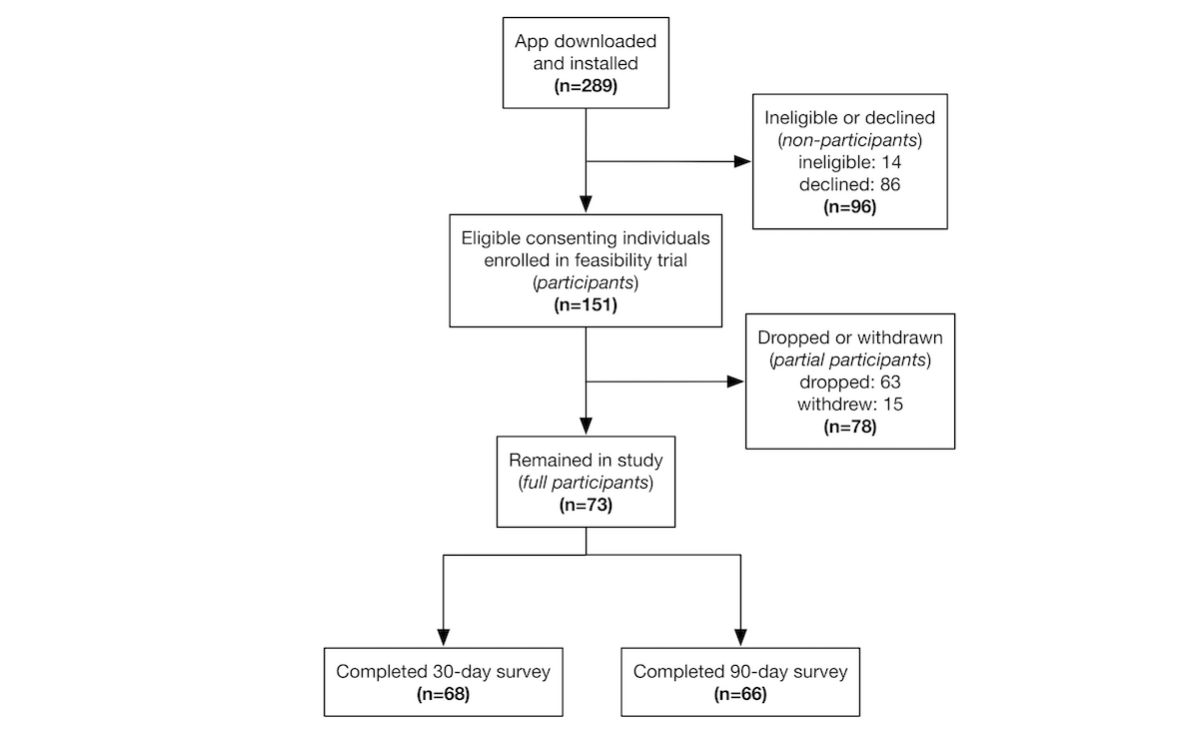
Flow of participants through the See Me Smoke-Free (SMSF) feasibility trial.

Recruitment of participants into the feasibility trial is illustrated in Figure 2. Of the total of 289 individuals who installed the SMSF app during the recruitment period, 251 completed the registration profile. Of these, 86 individuals chose not to enroll, and 14 were dropped because of exclusion criteria. A total of 151 eligible individuals consented to study participation and were enrolled, of whom 15 requested to be withdrawn from the study, and 63 were dropped because of inactivity, yielding 78 partial participants and 73 full participants. The median time until dropout because of either withdrawal-request or inactivity was 33 days, with a median time until withdrawal-request of 23.5 days (range: 15-48 days; n=15) and median time until dropout because of inactivity of 34 days (range: 4-82 days; n=63).

Baseline characteristics of study participants and nonparticipants are summarized in Table 1. Participants had significantly higher likelihood of female gender and of smoking in the 30 days before enrollment than nonparticipants. Whereas a larger proportion of participants than nonparticipants set a quit date at baseline, and participants smoked a higher mean number of cigarettes per day and paid a higher average price for cigarettes, these differences were not statistically significant. Participants were more likely to have been referred to SMSF by Google or the Google Play Store than nonparticipants, but other sources of referral did not differ significantly.

**Table 1:** Baseline characteristics from registration profile responses for all users who completed a profile; study participants and nonparticipants. Test results are for null hypotheses of no mean difference between participants and nonparticipants.

Profile characteristics		All users (N=247)	Participants (n=151)	Nonparticipants (n=96)	*P* value
		Mean (SD^a^) or n (%)	Mean (SD) or n (%)	Mean (SD) or n (%)	
Gender: female, n (%)		243 (98)	151 (100)	92 (96)	.02^b^
Smoked in previous 30 days, n (%)		237 (96)	151 (100)	86 (90)	<.001^b^
Set quit date at baseline, n (%)		198 (80)	127 (84)	71 (74)	.07^b^
Cigarettes smoked on a typical day, mean (SD)		16.1 (10.6)	16.6 (11.9)	15.2 (8.1)	.27^c^
Amount paid for a pack of cigarettes in US $, mean (SD)		6.61 (2.80)	6.70 (2.77)	6.47 (2.86)	.54^c^
**Referred to SMSF**^d^ **by**^e^**, n (%)**					
	Facebook	29 (12)	19 (13)	10 (10)	.69^b^
	Google or Google Play Store	78 (32)	56 (37)	22 (23)	.02^b^
	Friend or family	26 (11)	14 (9)	12 (13)	.52^b^
	Other	135 (55)	79 (52)	56 (58)	.36^b^

^a^SD: standard deviation.

^b^Fisher exact test.

^c^*t* test (2-tailed).

^d^SMSF: See Me Smoke-Free.

^e^Totals may exceed 100%, as users could provide more than one response.

Age and race data were collected only for participants, and one partial participant did not complete the baseline survey; all other baseline variables of interest were complete, and full and partial participants reported similar baseline characteristics. Age was similar among full and partial participants (full: mean=39.1 years, standard deviation [SD]=13.1 years; partial: mean=36.9 years, SD=11.4 years; *t* test: *P*=.29). Racial composition was also similar among full and partial participants (white: 73% [53/73] and 74% [57/77], respectively; African American: 16% [12/73], 12% [9/77]; Asian: 1% [1/73], 3% [2/77]; multiracial: 4% [3/73], 4% [3/77]; Native American: 0% [0/73], 1% [1/77]; other: 6% [4/73], 7% [5/77]; Fisher exact test: *P*=.94). Full and partial participants had moderate and similar concerns about weight gain (full: mean=3.1, SD=1.1; partial: mean=2.9, SD=1.2; on a scale from 1 to 5; *t* test: *P*=.49) and reported high smoking dependence, with 52 (71%, 52/73) full and 63 (82%, 63/77) partial participants reporting that they smoke within 30 min of waking (*t* test of reported dependence on continuous scale: *P*=.47). Most participants reported using mental imagery only infrequently at baseline, with 42 (58%, 42/43) full and 38 (49%, 38/78) partial participants reporting no use of mental imagery during the previous week (*t* test of reported mental imagery use on continuous scale: *P*=.76). Full and partial participants set quit dates at baseline at similar rates (Fisher exact test: *P*=.83) and reported similar numbers of cigarettes smoked at baseline (*t* test: *P*=.54) and amounts paid for cigarettes (*t* test: *P*=.10).

### Participation and Usage

On the basis of group-level usage data from Google Analytics, participants and nonparticipants opened the app and updated quit dates at the same average rate (Table 2), but participants started and completed audio files at significantly higher rates. Relative usage patterns of full versus partial participants could not be distinguished using the Google Analytics data. An in-depth comparison of daily assessment response rates between full versus partial participants was beyond the scope of this analysis; however, a total of 123 participants completed the daily assessment at least once.

Among these respondents (n=123), the daily assessment was completed on an average of 21.2 days (SD=26.6; range: 1-118). In response to the prompt “overall, please rate your cravings today,” users selected “none” on an average of 21 days (SD=25.2; range: 1-87), they selected “few” on an average of 9.2 days (SD=11.1; range: 1-63), they selected “several” on an average of 5.9 days (SD=9.6; range: 1-70), they selected “many” on an average of 3.9 days (SD=4.07, range: 1-21), and they selected “very many” on an average of 4.9 days (SD=9.2, range: 1-48). In response to the prompt “Did you smoke today, even a puff?” users selected “yes” on an average of 18 days (SD=23.7; range: 1-115), and they selected “no” on an average of 8.8 days (SD=13; range: 1-61).

### Participant Characteristics and Usage

Full participants listened to audio files on an average of 30.2 times (SD=29.4; range: 0-109) and answered questions on an average of 33.4 days (SD=29.1; range: 0-109). The use of negative binomial regression was supported by the highly right-skewed distributions of both usage variables, the significantly improved fit relative to Poisson models, and unremarkable residual plots. Usage outcomes were highly positively correlated (Pearson correlation ρ=.77). Whereas examined baseline characteristics were not significantly associated with extent of app usage (Table 3), results suggested higher relative usage with increasing age and non-white race and lower usage with increasing weight concern. Increased smoking dependence and use of mental imagery were not associated with consistent trends in app usage. Results did not differ meaningfully between unadjusted and adjusted models. Reanalysis after removal of influential observations reversed the association between race and audio completion and suggested a stronger relationship between increased smoking dependence and daily questions answered. No other estimates were highly sensitive to influential observations.

**Table 2:** Comparison of app usage outcomes from Google Analytics for participants and nonparticipants. Total: Aggregate count. Mean: Average per user. RR: rate ratio (exact Poisson test), with test for equal rates. Counts of audio files started and completed include both repeated usages of the same files and usages of different files by an individual.

Usage outcome	Participants (n=151)	Nonparticipants (n=96)	RR^a^ (95% CI)	*P* value
	Total (mean)	Total (mean)		
Opening the app	2912 (19.3)	1895 (19.7)	0.98 (0.92-1.04)	.43
Updated a quit date	29 (0.19)	18 (0.19)	1.02 (0.55-1.96)	>.99
Audio files started	2725 (18.0)	1625 (16.9)	1.07 (1.00-1.13)	.04
Audio files completed	2288 (15.2)	1315 (13.7)	1.11 (1.03-1.18)	.003

^a^RR: rate ratio.

**Table 3:** Associations between baseline characteristics and usage outcomes. App-based analytics for full trial participants (n=73) over the study period, as incidence rate ratios (IRR); results of sensitivity analysis, where influential observations are removed, are included.

Characteristic		Daily questions answered, IRR^a^ (95% CI)				Audio files completed, IRR (95% CI)		
		Unadjusted	Adjusted^b^	Influential observations removed^c^	Unadjusted		Adjusted	Influential observations removed
Age (per 10 years)		1.05 (0.87-1.26) *P*=.64	1.07 (0.89-1.30) *P*=.46	1.07 (0.89-1.30) *P*=.46, n=73	1.15 (0.96-1.38) *P*=.13		1.18 (0.98-1.42) *P*=.07	1.26 (1.05-1.51) *P*=.01, n=70
**Race**								
	White	Ref	Ref	Ref	Ref		Ref	Ref
	Other	1.16 (0.69-2.06) *P*=.59	1.25 (0.71-2.29) *P*=.43	1.25 (0.71-2.29) *P*=.43, n=73	1.02 (0.61-1.77) *P*=.95		1.23 (0.71-2.20) *P*=.44	0.86 (0.50-1.52) *P*=.44, n=70
**Time to smoke after waking**		*P*=.57	*P*=.72	*P*=.39	*P*=.18		*P*=.67	*P*=.17
	<5 min	0.90 (0.31-2.16)	0.92 (0.31-2.33)	1.53 (0.48-4.04) n=71	0.69 (0.26-1.60)		0.81 (0.30-1.96)	0.90 (0.31-2.22) n=68
	5-30 min	1.29 (0.46-3.02)	1.28 (0.46-2.99)	2.06 (0.67-5.17) n=71	1.26 (0.48-2.85)		1.19 (0.45-2.69)	1.62 (0.58-3.83) n=68
	31-60 min	0.90 (0.30-2.32)	0.90 (0.30-2.37)	1.26 (0.38-3.50) n=71	0.82 (0.29-2.04)		0.89 (0.32-2.20)	0.92 (0.31-2.44) n=68
	>60 min	Ref	Ref	Ref	Ref		Ref	Ref
Concern about weight gain^d^		0.89 (0.71-1.12) *P*=.32	0.91 (0.72-1.15) *P*=.43	0.88 (0.69-1.11) *P*=.25, n=68	0.83 (0.66-1.05) *P*=.09		0.85 (0.68-1.06) *P*=.13	0.78 (0.62-0.97) *P*=.02, n=69
Mental imagery use^e^		0.93 (0.76-1.16) *P*=.47	0.93 (0.76-1.17) *P*=.50	0.87 (0.69-1.11) *P*=.19, n=71	0.98 (0.81-1.22) *P*=.85		1.00 (0.83-1.23) *P*=.78	0.84 (0.69-1.05) *P*=.09, n=67

^a^IRR: incidence rate ratios.

^b^Model adjusted for age and race.

^c^n: number of observations remaining after exclusion of influential observations.

^d^1=low to 5=high.

^e^1=Never to 5=More than 30 min.

### App Usage and Smoking Cessation

Among 68 full participants who completed the 30-day survey, 7- and 30-day self-reported smoking abstinence was 37% (25/68) and 21% (14/68), respectively. Among 66 full participants who completed the 90-day survey, 7-, 30-, and 90-day self-reported smoking abstinence was 47% (31/66), 32% (21/66), and 15% (10/66), respectively. Cessation outcomes at the 30-day survey were highly positively correlated (Pearson correlation ρ=.77), but cessation outcomes at the 90-day survey and between 30- and 90-day surveys were less highly correlated (ρ=.43-.73). As our study design (within subjects) was focused on looking only at participants who actually used the app, usage data are not available for partial participants.

The data suggested that app usage may have a nonlinear relationship with smoking cessation, but model comparisons supported use of untransformed usage variables in all final analyses. Among full participants, odds ratios for most associations between app usage and smoking cessation were greater than one (Table 4), and some were statistically significant, suggesting a positive association between app usage and smoking cessation. Missing cessation outcomes were not imputed as missingness was low among full participants (7% [5/73] and 10% [7/73] for 30- and 90-day survey outcomes, respectively; partial participants lacked outcome data by definition and were not considered missing for this purpose). Adjusted models had moderate discriminatory ability, with *C* indices ranging from 0.68 to 0.78 (results not shown). Results were similar between unadjusted and adjusted models, though adjusted models yielded larger effect sizes. With the exception of 7-day smoking cessation at the 30-day survey, reanalysis after exclusion of influential observations also suggested stronger associations between app usage and smoking cessation than the primary analysis.

**Table 4:** Associations between app usage during the first 30 days and smoking cessation outcomes from 30- and 90-day surveys for full trial participants. To provide meaningful comparisons, estimates are given for the third versus first quartiles of the main exposures. Influential observations removed: Results of sensitivity analyses (adjusted models).

Self-reported smoking abstinence		Complete audio listens, OR^a^ (95% CI)				Days answering questions, OR (95% CI)		
		Unadjusted	Adjusted^b^	Influential observations removed^c^	Unadjusted		Adjusted	Influential observations removed
**30-day survey (n=68)**							
	7 days	1.57 (0.62-3.95) *P*=.34	1.78 (0.61-5.23) *P*=.29	1.51 (0.35-6.48) *P*=.58, n=63	1.28 (0.49-3.31) *P*=.62		1.63 (0.52-5.14) *P*=.40	1.21 (0.26-5.62) *P*=.81, n=63
	30 days	1.31 (0.44-3.87) *P*=.63	1.33 (0.37-4.69) *P*=.66	4.35 (0.36-52.08) *P*=.25, n=61	0.79 (0.25-2.52) *P*=.69		0.86 (0.22-3.38) *P*=.83	0.87 (0.10-7.63) *P*=.90, n=61
**90-day survey (n=66)**							
	7 days	2.84 (1.05-7.66) *P*=.04	3.04 (1.07-8.67) *P*=.04	4.19 (1.36-12.97) *P*=.01, n=62	2.54 (0.96-6.67) *P*=.06		3.17 (1.07-9.38) *P*=.04	6.27 (1.62-24.27) *P*=.01, n=62
	30 days	2.37 (0.87-6.46) *P*=.09	3.06 (1.00-9.35) *P*<.05	9.12 (1.99-41.79) *P*<.01, n=61	1.79 (0.67-4.79) *P*=.25		2.55 (0.82-7.95) *P*=.11	3.87 (1.09-13.73) *P*=.04, n=64
	90 days	1.93 (0.55-6.77) *P*=.30	2.91 (0.63-13.43) *P*=.17	3.61 (0.83-15.80) *P*=.09, n=56	1.03 (0.29-3.70) *P*=.96		1.65 (0.36-7.65) *P*=.52	N/A^d,e^

^a^OR: odds ratio.

^b^Models adjusted for age, race, mental imagery, weight concern, and tobacco dependence.

^c^n: number of observations remaining after exclusion of influential observations.

^d^N/A: not applicable.

^e^Results were unreliable as nearly all (9 of 10) individuals with reported smoking cessation were flagged as influential.

## Discussion

### Principal Findings

The results of this study supported many hypothesized associations in the SMSF trial. Participants were significantly more likely to report that they had smoked during the 30 days before enrollment and to report female gender, both of which were requirements of study enrollment. These results suggest that the app may have appeal beyond the target group. One aim of this study was to explore how the sample of participants compared with users who may or may not have met the inclusion criteria and how other people who downloaded the app (eg, men and those not ready to quit) used the app. As participants were more likely than nonparticipants to have learned about SMSF from Google or the Google Play Store, it is possible that participants were more highly motivated to quit smoking and to have actively sought out cessation-related tools or products. Results also suggested possible associations between usage and both age and weight concern, suggesting a need for further investigation to understand the relationship between these variables. Age has been observed to be a predictor of usage of smoking cessation websites [14]; however, associations with mobile phone–based cessation programs have been mixed, as noted by Zeng et al [10]. Regarding weight concern, the SMSF app was targeted at women smokers who were concerned about weight gain, which is why it includes diet and physical activity components aimed at reducing weight gain while quitting smoking. Study participants did not open the app more frequently than nonparticipants but did start and complete audio files at a significantly higher rate. Conclusions about the clinical meaningfulness of these differences cannot be made because the same outcome measures for participants and nonparticipants were not collected. A positive dose-response relationship to the number of audio files listened to, and smoking abstinence, has been identified by an earlier analysis of the SMSF program [9]. Most of the analyses suggested that participants who used the app more frequently were also more likely to quit smoking, consistent with the findings of Gordon et al [9], who reported significant increases in 7- and 30-day self-reported smoking abstinence during follow-up along with improvements in certain aspects of physical activity and diet.

The contribution of SMSF to the field of mHealth as an innovative imagery-based, multi-behavioral intervention has been discussed in earlier publications [7-9]. Uncertainty often surrounds the extent to which the use of digital interventions determines desired health outcomes [5]. A review by Donkin et al [15] discussed how the most appropriate metrics of usage may differ between different types of interventions and how in-depth analysis of usage can help understand which metrics are most associated with effectiveness. Additionally, user characteristics can strongly influence the effectiveness of digital interventions [6]. This study sought to determine characteristics of users in the SMSF feasibility trial, how these characteristics might be related to app usage, and to assess whether there is preliminary evidence that app usage is associated with self-reported smoking abstinence.

### Strengths and Limitations

Limitations of the SMSF feasibility study have been discussed by Gordon et al [9]. Potential sources of bias include the lack of a control group and the significant dropout rate. Among those who used the app for the duration of the study and responded to follow-up measures, app use was positively associated with cessation. Retention on this study is comparable with other mHealth studies, which frequently have high attrition rates [16-19]. The high attrition inherent in many mHealth studies is offset by the broad reach and dissemination potential of mHealth intervention programs. Participants were recruited from a self-selected pool of individuals who responded to project news coverage and social media promotions, raising the risk of selection bias. Respondents could have differed from the broader target population of female smokers, for example, by being more highly motivated to quit and more inclined to engage in mHealth interventions. The majority of full participants self-identified as white, possibly limiting generalizability to other populations or indicating a need to target the app to less represented groups [20]. Furthermore, SMSF is available exclusively on Android phones, potentially narrowing the user base by socioeconomic status [15]. Despite these limitations, the SMSF trial had several strengths, including the analysis of multiple independent sources of usage data. Whereas some results were sensitive to a small number of influential participants, an additional strength of the study was the general robustness of most estimates. Important additional factors (variables, interactions, or nonlinear relationships) determining app usage or smoking cessation may remain unidentified, but the large effect sizes estimated for most associations between app usage and cessation outcomes are promising and support more extensive evaluation of SMSF efficacy. We note that as a feasibility study, this trial’s primary objective was not to detect intervention effects, so results should be interpreted cautiously.

### Conclusions

This study suggests high potential efficacy of the SMSF app, as increased usage was generally associated with higher self-reported smoking abstinence. As a one-arm feasibility trial, a causal relationship between app usage and improved smoking cessation cannot be demonstrated, and the study was not powered to identify significant associations. A planned SMSF controlled trial should provide additional evidence with which to judge the app’s efficacy as an intervention tool and afford greater statistical power and may be able to examine additional factors affecting app usage and smoking cessation.

## Abbreviations

IRR: incidence rate ratio
LRTs: likelihood ratio tests
mHealth: mobile health
OR: odds ratio
RR: rate ratio
SD: standard deviation
SMSF: See Me Smoke-Free
